# The effect of L-carnitine in reactive oxygen species reduction and apoptotic gene expression in mice after cyclophosphamide: An experimental study

**DOI:** 10.18502/ijrm.v22i8.17262

**Published:** 2024-10-14

**Authors:** Majid Almasi, Golnaz Shafiei, Hossein Nikzad, Mohammad Karimian, Ghazaleh Moshkdanian

**Affiliations:** ^1^Gametogenesis Research Center, Kashan University of Medical Sciences, Kashan, Iran.; ^2^Anatomical Sciences Research Center, Institute for Basic Sciences, Kashan University of Medical Sciences, Kashan, Iran.; ^3^Department of Molecular and Cell Biology, Faculty of Basic Sciences, University of Mazandaran, Babolsar, Iran.

**Keywords:** Carnitine, Cyclophosphamide, Apoptosis, Genes, Reactive oxygen species.

## Abstract

**Background:**

Cyclophosphamide (CP), a utilized anticancer drug, is known to cause infertility in women. However, L-carnitine (LC), an antioxidant, has been shown to offer protective benefits against infertility.

**Objective:**

This study aimed to evaluate the levels of reactive oxygen species (ROS) and apoptotic gene expression in mice treated with CP and LC.

**Materials and Methods:**

24 NMRI female mice (6–8 wk, 30 ± 5 gr) were divided into 4 groups: control group: received normal saline intraperitoneal (IP) injection for 10 days; CP group: received 75 mg/kg of CP as a single IP on the 10 ${\;}^{\text{th}}$ day of the experiment; LC group: received 200 mg/kg of LC IP for 10 days; LC+CP group: received LC for 10 days and CP single IP injection on the 10 ${\;}^{\text{th}}$ day of the experiment. After 10 days, mice were superovulated. The oviducts were then removed, and the oocytes of each group were collected for evaluating apoptotic gene expression B-cell lymphoma 2(*Bcl2*), *Bcl2*-associated X(*Bax*), and *Caspase3* via real-time polymerase chain reaction and intracellular ROS levels by dichloro-dihydro-fluorescein diacetate fluorescence staining.

**Results:**

Data revealed that LC in the LC+CP group significantly increased *Bcl2* gene expression (p = 0.01), and decreased *Bax* and *Caspase3* gene expression compared to the CP group (p = 0.03, p = 0.04). LC decreased the ROS level in the LC+CP group compared to the CP group (p < 0.001).

**Conclusion:**

Findings suggest that LC can scavenge the ROS caused by CP and modulate the apoptotic pathway via downregulating the *Bax* and *Caspase3* genes and upregulating the *Bcl2* gene in oocytes of mice exposed to CP.

## 1. Introduction 

The surprising competence of cancer chemotherapy has recently resulted in significantly improved survival and life expectancy in cancer patients. Thereby, young cancer patients have been considerably interested in preserving their fertility after cancer treatment. On the other hand, all the chemotherapy medicines, no doubt, cause harmful effects on reproductive performance. Among different drug types, alkylating agents are generally coupled with reproductive issues (1). Cyclophosphamide (CP), an “alkylating agent”, has been extensively used in anticancer drugs (2). Even though cancer patients who received the CP treatment experienced oocyte damage, primordial follicle destruction, and meiotic spindle abnormalities. It also adversely affected their offspring outcomes and fertility (3, 4).

Thus, evaluating and preserving the quality of fertility after CP exposure is crucial (5). Accumulating studies declared that reactive oxygen species (ROS) generated by CP is considered the most pivotal event that leads to infertility; however, the mechanisms contributing to CP causing oocyte damage remain unclear. A summary evaluation of the published studies indicates that excessive ROS generation plays an important role in activating the apoptotic pathways via various undesirable effects such as membrane and DNA damage, lipid peroxidation, spindle morphology, and microtubule arrangement (6).

Scientists demonstrated that antioxidant supplementation is associated with overcoming harmful effects caused by ROS and alleviating apoptosis in oocytes (7, 8). L-carnitine (LC) is a small, highly polar, water-soluble quaternary amine essential for fat metabolism. The active form of carnitine, LC (3-hydroxy-4-N-trimethyl amino butyrate), is primarily synthesized from the amino acids lysine and methionine in the liver (9). As an antioxidant, LC neutralizes free radicals, particularly superoxide anions, safeguarding cells from oxidative damage-induced apoptosis and enhancing the quality of oocytes and embryos (10). Previous research has shown that LC protects female reproductive health by mitigating oxidative stress in oocytes, thereby improving their quality (11, 12). In sheep models, supplementing with LC during in vitro maturation mitigated embryo toxicity caused by oxidative stress. This was achieved by reducing intracellular ROS levels and boosting intracellular glutathione levels, which consequently enhanced the developmental potential of oocytes and embryos and altered the gene expression levels for antioxidant enzymes (9).

As to scientific evidence, the correlation between CP and the apoptotic pathway in oocytes is not fully understood, and the restriction of generated ROS from chemotherapy drugs is the most important counteraction. Also, there is not enough data available that indicates the effects of LC after exposure of oocytes to CP. Therefore, this study has been initiated to examine the possible protective effects of LC against oocyte injury, and to investigate the apoptosis pathway in oocytes after exposure to CP.

## 2. Materials and Methods

### Animal preparation 

In this experimental study, 24 NMRI female mice (6–8 wk, 30 ± 5 gr) were purchased from the Research Center of Kashan University of Medical Sciences (Kashan, Isfahan, Iran). After 7 days of adapting, animals were maintained to access the controlled conditions of temperature (25 ± 2 C), good ventilation, and lighting (12 hr light/dark cycle).

### Experimental design 

**Figure 1:**
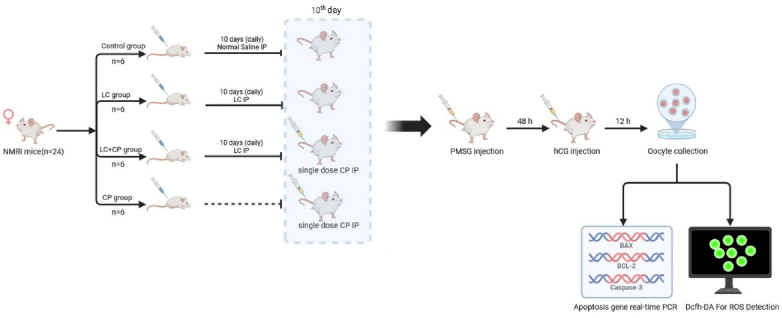
The flow chart of this study. *Bax*: *Bcl2*-associated X, LC: L-carnitine, CP: Cyclophosphamide, IP: Intra peritoneal, PMSG: Pregnant mare serum gonadotrophin*,* HCG: Human chorionic gonadotropin, PCR: Polymerase chain reaction, DCFH-DA: 2',7'-dichlorofluorescein diacetate, ROS: Reactive oxygen species.

The sample size was selected based on the previous study (Figure 1) (13). The female NMRI mice were divided into 4 groups randomly (n = 6/group) and housed in polycarbonate cages (n = 3/cage). Design groups are in the following:

Control group: Received 2.5 ml/kg normal saline intraperitoneal (IP) injection for 10 days.

LC group: Received 200 mg/kg of LC (Sigma-Aldrich Co., St. Louis, MO, USA) IP injection for 10 days (10, 14).

LC+CP group: Received 200 mg/kg IP injection for 10 days and CP (Endoxan, Baxter Oncology GmbH, Halle, Germany) 75 mg/kg single IP injection on the 10 ${\;}^{\text{th}}$ day of the experiment.

CP group: Received 75 mg/kg of CP as a single IP injection on 10 ${\;}^{\text{th}}$ day of the experiment (10).

### Oocyte collection

Female mice were superovulated by IP injection of 8 IU pregnant mare serum gonadotropin (Sigma-Aldrich, China), after 10 days injection of LC, CP, or saline. Then, 48 hr later, a similar dose of human chorionic gonadotropin (Pregnyl; Organon, Germany) was also given to the females.

Mice were sacrificed by cervical dislocation 13.5 hr later and oviducts were flushed in Ham's F10 media (Thermo Fisher Scientific, USA) under a stereomicroscope (Olympus SZX9, Japan), the cumulus cells were removed enzymatically using 0.3 mg/ml hyaluronidase (Sigma-Aldrich, USA) and only mature oocytes with homogeneous cytoplasm, normal perivitelline space, and zona pellucida were considered morphologically normal. Selected oocytes were incubated in media drops under liquid paraffin oil at 37 C, 5% CO_2_ incubator to be used in experiments (10, 14).

### RNA isolation and real-time polymerase chain reaction (RT-PCR)

The expression levels of *Bcl2*-associated X (*Bax*), *Caspase3*, and B-cell lymphoma 2 (*Bcl2*) genes in oocytes (n = 60/each) were quantified using RT-PCR. RNA was extracted from each group utilizing the RNeasy Micro kit (74004, Qiagen, USA), adhering to the manufacturer's guidelines (10). Subsequently, the isolated RNA served as a template for cDNA synthesis, performed with the Prime Script ${\;}^{\text{TM}}$ RT reagent kit (Takara Bio Inc., Shiga, Japan), using 500 ng of total RNA under the provided protocol. The RT-PCR analysis involved 2 µl of the cDNA, combined with the SYBR green master mix (BioFACT, Korea), in a final volume of 10 µl. This reaction was executed on the IQ5 RT-PCR system (Bio-Rad, Germany). Primer sequences employed for each gene are detailed in table I. To normalize the expression of long non-coding RNAs, glyceraldehyde 3-phosphate dehydrogenase (*GAPDH*) mRNA levels were used as a reference, and the relative changes were calculated using the 2^-ΔΔc^ method.

### Measurement of ROS in oocytes 

To quantify the relative levels of ROS in oocytes, we employed the fluorescent dye 2',7'-dichlorofluorescein diacetate (DCFH-DA, 2 µM, Sigma, D6883, USA). A group of 5–10 oocytes were incubated for 20 min in Ham's F10 medium enriched with DCFH-DA (2 µM) at 37 C in darkness. Postincubation, the oocytes were rinsed with Ham's F10 medium supplemented with 10% human serum albumin (N6908, Sigma, USA) and then placed on glass slides. Fluorescence measurements were conducted using a Nikon Eclipse Ti fluorescence microscope (533450, Japan), utilizing excitation and emission filters with wavelengths of 450–490 nm and 520 nm, respectively. The fluorescence intensity, indicative of ROS levels, was quantified for each oocyte using Image Quant software (TotalLab Quant, UK) (15).

**Table 1:** List of specific primers used in real-time polymerase chain reaction assay


**Gene name**	**Accession no.**	**Designed oligonucleotide (5 '→ 3 ' )**	**Product size (bp)**
***Bax***	NM_138764.5	F: TTGCTACAGGGTTTCATCCAG R: CCAGTTGAAGTTGCCATCAG	246
***Caspase3***	NM_004346.4	F: AAAGACCATACATGGGAGC R: CGAGATGACATTCCAGTGCT	138
***Bcl2***	NM_016993.1	F: GGGAGAGTCAACAGGGAGA R: CTTCAGAGACAGCCAGGAGA	177
***GAPDH***	MH759770	F: AACTTTGGCATTGTGGAAGG R: ACACATTGGGGGTAGGAACA	233
*Bax*: *Bcl2*–associated X, *Bcl2*: B-cell lymphoma 2, *GADPH*: Glyceraldehyde 3-phosphate dehydrogenase			

### Ethical considerations 

The procedures were approved by the Kashan University of Medical Sciences, Kashan, Iran (Code: IR.KAUMS.MEDNT.REC.1396.45). The animal experimental protocols were supported by the Iranian Council's guidelines for the Use and Care of Animals.

### Statistical analysis

Each experiment was performed in triplicate, and statistical analysis was conducted using GraphPad Prism 8 (San Diego, CA, USA) and SPSS software version 16.0 (SPSS Inc., Chicago, IL, USA). A one-way ANOVA test (comparison between 3 or more experimental groups) was performed followed by post hoc Tukey's test. The results were expressed as the mean ± standard deviation. The mean differences between groups for ROS and gene expression levels were compared using analysis of variance (ANOVA), followed by post hoc Tukey's test. P < 0.05 were considered statistically significant.

## 3. Results

### The effect of LC and CP on changes in the expression of apoptosis genes (*Bax*, *Caspase3*, *Bcl2*) 

To investigate whether apoptosis genes are up- or downregulated among studied groups (control, LC, LC+CP, CP), we monitored the relative change in mRNA expression of proapoptotic genes (*Bax* and *Caspase3*) and antiapoptotic gene (*Bcl2*) with RT-PCR.

**Figure 2:**
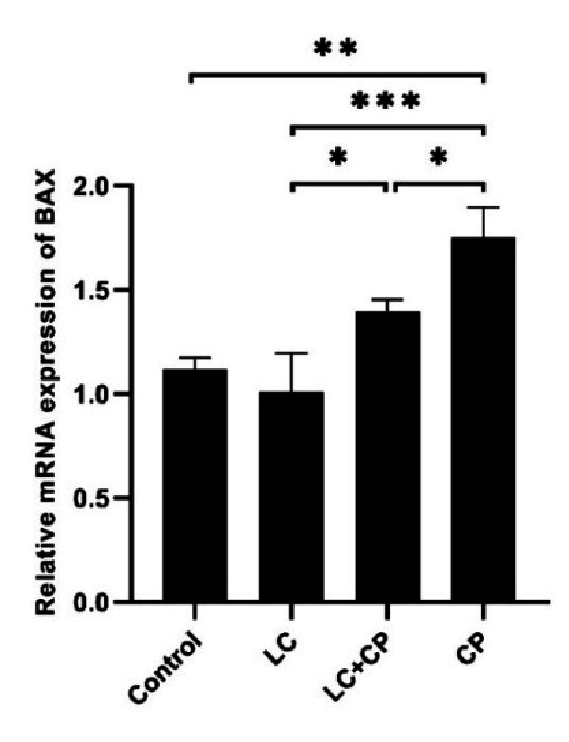
Downregulation of *Bax* gene expression in mouse oocytes following treatment with LC compared with the CP group. RT-PCR analysis of *Bax* mRNA. *P < 0.05, **P < 0.01, ***P < 0.001. *Bax*: *Bcl2-*associated X, LC: L-carnitine, CP: Cyclophosphamide, RT-PCR: Real-timepolymerase chain reaction.

**Figure 3:**
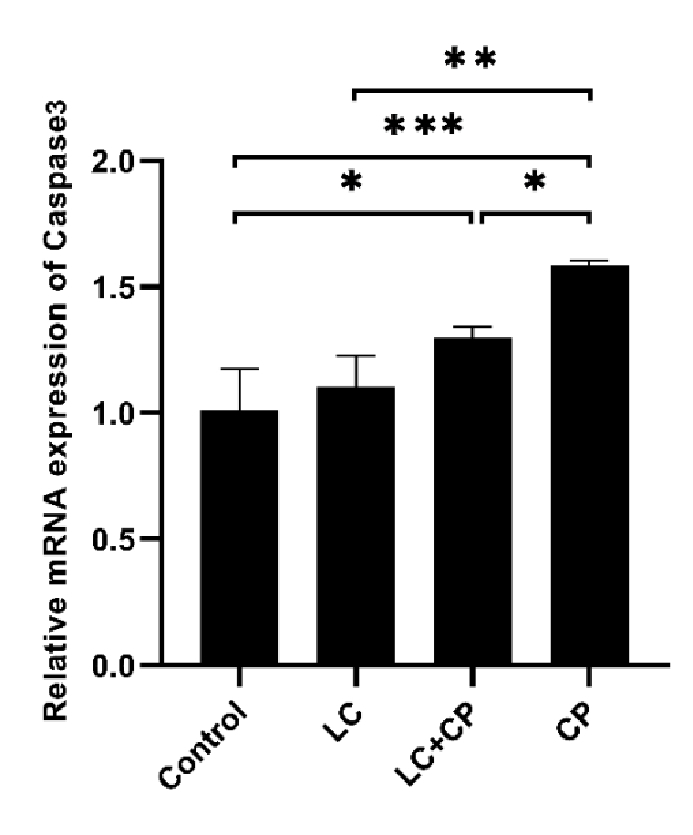
Downregulation of *Caspase3* gene expression in mouse oocytes following treatment with LC compared with the CP group. RT-PCR analysis of *Caspase3* mRNA. *P < 0.05, **P < 0.01, ***P < 0.001. LC: L-carnitine, CP: Cyclophosphamide, RT-PCR: Real-time polymerase chain reaction.

As shown in figures 2 and 3, the expression of the *Bax* and *Caspase3* genes significantly upregulated in the CP group in comparison to the LC+CP (p = 0.03, p = 0.04), LC (p < 0.001) and control (p < 0.001) groups, respectively. The lowest significant expression of the *Bax* gene was found in the LC group in comparison to the LC+CP (p = 0.02) group. Interestingly, the expression level of the *Bax* gene in the LC+CP group was nonsignificant in comparison to the control group (Figure 2). The LC+CP group had a significantly (p = 0.04) higher expression of *Caspase3* genes than control group (Figure 3).

**Figure 4:**
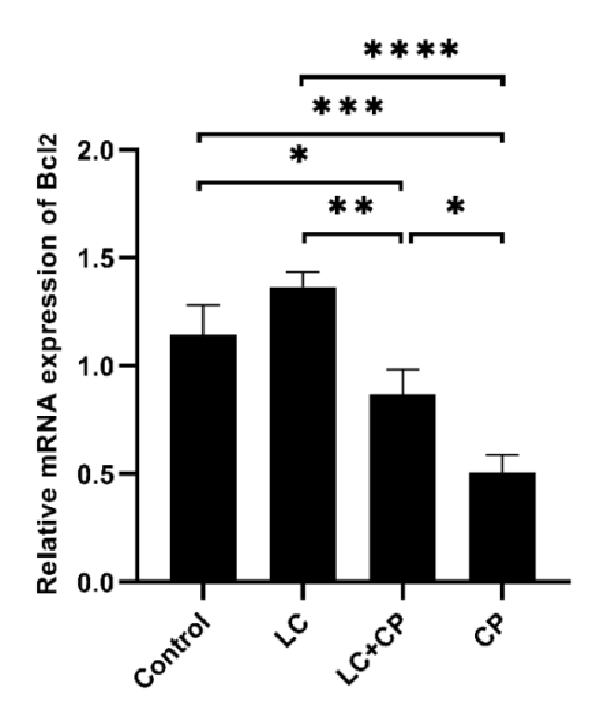
Upregulation of *Bcl2* gene expression in mouse oocytes following treatment with LC compared with the CP group. RT-PCR analysis of *Bcl2* mRNA. *P < 0.05, **P < 0.01, ***P < 0.001, ****P < 0.000. *Bcl2*: B-cell lymphoma 2, LC: L-carnitine, CP: Cyclophosphamide, RT-PCR: Real-time polymerase chain reaction.

As depicted in figure 4 a significant upregulation in *Bcl2* expression was observed in the LC group compared to LC+CP (p < 0.001) and CP (p < 0.001) groups. In addition, a significant decrease was observed in the expression level of *Bcl2* in the CP group in comparison to the LC+CP (p = 0.01), and control (p < 0.001) groups. Moreover, the expression of the *Bcl2* gene in the LC+CP group significantly decreased compared to control group (p = 0.04) (Figure 4).

### The effect of LC and CP on the intracellular ROS level

**Figure 5:**
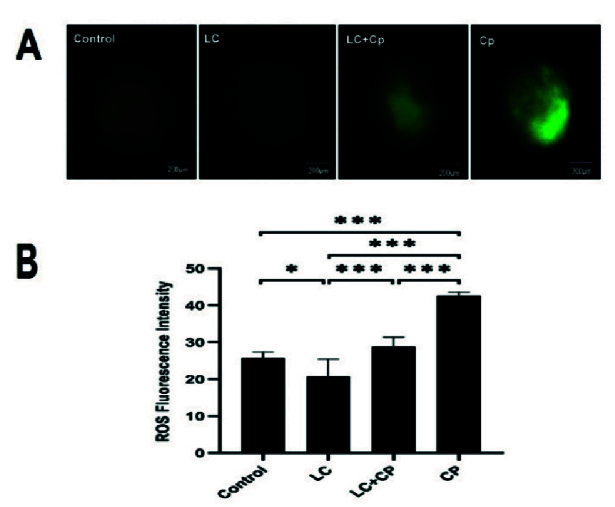
Effect of LC treatment on ROS concentration in mouse oocytes. A) The rate of intracellular ROS was shown by the DCFH-DA fluorescence intensity (green) in all control, LC, LC+CP, and CP studied groups. B) Measures of intensity of staining. *P < 0.05, ***P < 0.001. LC: L-carnitine, ROS: Reactive oxygen species, DCFH-DA: 2',7'-dichlorofluorescein diacetate, CP: Cyclophosphamide.

Oocyte intracellular ROS levels were assessed using a DCFH-DA fluorescence assay based on the protocol previously described (15). As described in figure 5, LC could markedly diminish the generation of ROS in the LC group in comparison to the CP (p < 0.001), LC+CP (p < 0.001), and control (p = 0.01) groups. Also, administration of LC significantly decreased the ROS level in the LC+CP group compared to that of the CP group (p < 0.001). Furthermore, a significant (p < 0.001) increase in ROS level was detected in the CP group compared to control group (Figure 5), no statistical difference was observed between control and LC+CP groups.

## 4. Discussion

Our findings indicated a direct correlation between increasing the production of ROS and activating apoptotic pathways following the treatment with CP. We have shown notable alterations in ROS level and the expression of pro-apoptotic genes (*Bax* and *Caspase3*) and an anti-apoptotic gene (*Bcl2*) in the oocytes of mice exposed to CP group when compared to that of the other groups which can improve by LC. Many reports imply the adverse effect of CP on oocytes. However, the mechanisms presenting how the CP makes oocyte incompetency are still unknown (6).

In this study, we found the upregulation of gene expression of *Bax* and *Caspase3* and downregulation of gene expression of the *Bcl2* in the CP group in comparison to the other groups. This modification can trigger the apoptotic pathway associated with oxidative stress and the induction of ROS and reactive nitrogen species. This, in turn, can cause DNA damage, ultimately leading to the mitochondrial apoptotic pathway by reducing the levels of anti-apoptotic proteins (16). One study showed that the expression levels of *Bax* were elevated in the CP group. This elevation exerts a toxic effect by causing abnormal DNA base pairings, which leads to malformed and dysfunctional cells, resulting in irreversible damage to the ovary (17). So, CP as the most successful and frequently used anticancer drug can cause side effects on the ovaries and oocytes and eventually result in low fertility rates (3, 18). Also, CP can increase the apoptosis of granulosa cells, leading to the depletion of the ovarian reserve of primordial follicles and, consequently, ovarian function failure (19). Active metabolites of CP can prevent DNA synthesis and disrupt the antioxidant defense system of the ovary and ultimately, lead to excessive production of ROS (20).

Furthermore, the generated ROS within the mitochondria can induce lipid peroxidation in the mitochondrial membrane during CP metabolism. This can affect the mitochondrial membrane potential and lead to the release of cytochrome c, triggering apoptosis through the activation of *Caspase3* (21). The release of mitochondrial cytochrome c can be further enhanced by p53-induced *Bax* activation in a caspase-dependent manner (16).

Extracted data from this study showed that the injection of 75 ml/kg CP could significantly increase the level of ROS. In agreement with our results, previous studies have stated that injection of 120 mg/kg of CP yields a significant increase in oocyte intracellular ROS in mice. Yet, most studies on oxidative injury to oocytes focus on some major cellular functions, such as impaired mitochondrial function, the formation of meiotic spindle, configuration, and DNA damage (6, 22). Consequently, the detoxification of ROS might be impressive for improving the oocyte competence to preserve fertility in women treated with chemotherapy (7, 23). Since antioxidants scavenge free radicals and reduce ROS levels in the body (24), these may also be useful in minimizing the harmful effects of free radicals on the apoptotic signaling pathway. There are several reports declaring that both enzymatic and nonenzymatic antioxidants are used to reduce the level of ROS generation (23–25). They have revealed that LC has some properties similar to nonenzymatic antioxidants, although it lacks the activity to scavenge free radicals directly (26).

This compound is widely distributed in nature, especially in red meats and dairy, and can protect loads of cells from oxidative injury and apoptosis in primary neuronal cells as well as some non-neuronal cell lines (27, 28). Many articles have also indicated the protective effects of LC against the various chemotherapy drugs and radiotherapy in different organs and tissue (29–31), but based on our broad search not enough data were available that confirms the protective effect of LC against CP-induced damage and apoptosis in the oocyte.

Additionally, LC's role as an antioxidant is crucial in preventing the depletion of ATP reserves caused by the accumulation of ROS in follicles, which otherwise leads to diminished follicle quality (32, 33). Our result showed that treatment with 200 mg/kg LC in mice could significantly reduce the intracellular ROS accumulation in the LC group rather than that in the other groups. In confirmation of our results, studies suggested that LC can reduce the oxidative stress of cells by increasing the expression of antioxidant proteins such as superoxide dismutase 2 and improving the ability of the oocyte to reduce the probable damage (34, 35).

Likewise, treating oocytes with LC in culture can enhance their quality, potentially due to a decrease in granulosa cell apoptosis and an improvement in mitochondrial function. Additionally, it has been documented that administering LC can help mitigate delayed embryonic development, DNA fragmentation, and abnormal blastocyst formation resulting due to prolonged culture under high ROS conditions (11). Therefore, LC treatment might enhance follicle quality and improve pregnancy outcomes after in vitro fertilization and embryo transfer. Recent studies have highlighted the protective effects of LC on various organs like rat testicular toxicity (36), reducing oxidative stress in the rat brain (37) and cardiac injury in rats (38). LC shields miotic oocytes from damage induced by follicular fluid in women with mild endometriosis (39). It may inhibit apoptosis by enhancing the β-oxidation of fatty acids, thereby reducing their toxicity.

Moreover, data from our study described that LC could reduce the expression of pro-apoptotic genes (*Bax* and *Caspase3* genes) and significantly enhance the expression of the antiapoptotic gene (*Bcl2* gene) in the LC group. Interestingly, we found that using LC in the LC+CP group can significantly upregulate the expression of the antiapoptotic gene and down-regulate pro-apoptotic genes when compared with the CP group. Remarkably, the expression of the *Bax* gene in the LC+CP group was the same as that presented in the control group. LC has been shown to mitigate the effects of formalin and enhance *Bcl2* expression while decreasing *Bax* expression, suggesting its potential role in inhibiting apoptosis (40).

One study demonstrated that supplementing porcine cumulus oocyte complexes with LC, at a concentration of 0.5 mg/mL during in vitro maturation enhanced nuclear maturation. After parthenogenic activation, this supplementation led to an increased number of blastocysts with a reduced number of apoptotic cells, suggesting that the positive effects of LC might be linked to its antiapoptotic properties (34). This finding was confirmed by studies conducted on a mouse model. When mouse embryos were exposed to hydrogen peroxide or tumor necrosis factor (TNF), there was a decrease in blastocyst development rates accompanied by increased apoptosis. However, the introduction of LC at concentrations of 0.3 or 0.6 mg/ml significantly mitigated the detrimental effects of both hydrogen peroxide and TNF (34). The antiapoptotic and proliferative effects of LC during embryo development were supported by changes in the expression of apoptotic genes, such as *Bax, Bcl2,* and *Caspase3*. This was further confirmed by observations that LC could counteract the proapoptotic and antiproliferative effects of actinomycin D and TNF-alpha on cleavage-stage embryos (41). Furthermore, LC reduces apoptosis induced by mitochondria in both in vivo and in vitro conditions (42). A similar study showed that LC not only stabilizes mitochondrial membranes but also enhances the energy supply to the organelle and protects cells from apoptosis (43). Data from this study confirmed that LC can potentially decrease the pro-apoptotic gene expression in mice exposed to CP.

### Limitations

More research is necessary to clarify the molecular mechanisms underlying the function of LC to repair damaged oocytes and ovaries after chemotherapy by CP.

## 5. Conclusion 

LC can alleviate the adverse effects of CP chemotherapy, including intracellular ROS accumulation, and influence the apoptosis pathway in CP-exposed mice oocytes by decreasing the ROS level and the expression of *Bax* and *Caspase3* genes, while increasing the *Bcl2* gene expression.

##  Data availability

The data and materials supporting this study's findings are available from the corresponding author upon reasonable request.

##  Author contributions

Gh. Moshkdanian, H. Nikzad, M. Almasi, and G. Shafiei had full access to all of the data in the study and take responsibility for the integrity of the data and the accuracy of the data analysis. Concept and design Gh. Moshkdanian, H. Nikzad, and M. Almasi. Acquisition, analysis, or interpretation of data Gh. Moshkdanian, H. Nikzad, M. Karimian, M. Almasi, and G. Shafiei. Drafting of the manuscript: M. Almasi, G. Shafiei, and M. Karimian. Critical revision of the manuscript for important intellectual content: All authors. Statistical analysis: M. Almasi, G. Shafiei, and Gh. Moshkdanian. Supervision: Gh. Moshkdanian and H. Nikzad.

##  Conflict of Interest 

No conflict of interest.
